# CALPHAD-guided interlayer design for crack-free additive manufacturing of copper C18150 – Inconel 625 bimetallic structures

**DOI:** 10.1080/14686996.2025.2587393

**Published:** 2025-11-26

**Authors:** Liyi Wang, Luis Fernando Ladinos Pizano, Michael A. Klecka, Kelsay Neely, Wei Xiong

**Affiliations:** a Physical Metallurgy and Materials Design Laboratory, Department of Mechanical Engineering and Materials Science, University of Pittsburgh, Pittsburgh, PA, USA; b RTX Technology Research Center, East Hartford, CT, USA; c NASA Marshall Space Flight Center, Huntsville, AL, USA

**Keywords:** Wire arc additive manufacturing, phase separation, cracking susceptibility, extreme environments

## Abstract

Additive manufacturing (AM) of bimetallic structures combining copper alloys and Ni-based superalloys is critical for extreme environmental applications. However, interface cracking during fabrication persists due to thermophysical property mismatches. By implementing a CALPHAD-based ICME framework (CALPHAD: Calculation of Phase Diagrams; ICME: Integrated Computational Materials Engineering), we decode nonequilibrium solidification and phase stability to predict cracking susceptibility. Liquid phase separation emerges as the dominant mechanism, altering solute redistribution and thermal stress accumulation – a previously underexplored factor in bimetallic systems. Experiments using wire arc additive manufacturing (WAAM) validate model prediction: crack-free interfaces between C18150 and In625 require intermediate layers with 65 wt.% In625. This composition mitigates cracking with the lowest cracking susceptibility coefficient (CSC). Importantly, we establish a quantitative correlation between phase separation and CSC, proposing a way to analyze systems exhibiting these microstructural features. This work uses ICME methodologies by linking thermochemical modeling to process optimization, offering new principles for designing defect-resistant bimetallic components in extreme environments such as rocket engine nozzles.

## IMPACT STATEMENT

CALPHAD-based ICME framework reveals liquid phase separation drives bimetallic interface cracking, establishing a quantitative cracking susceptibility correlation to enable predictive defect-resistant design in functionally graded materials by additive manufacturing.

## GRAPHICAL ABSTRACT

**Figure:**
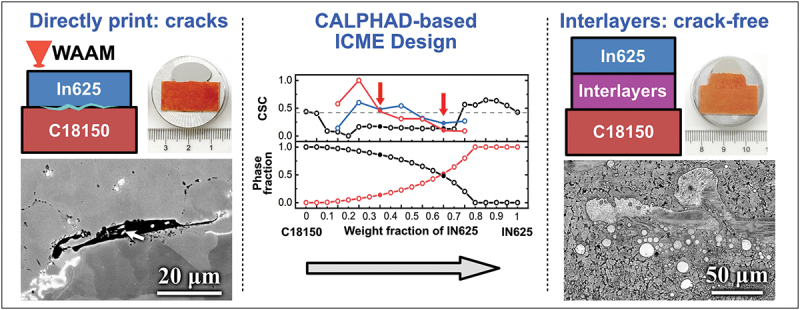

## 1. Introduction

The design of liquid rocket engine nozzles, particularly those employing regenerative cooling or channel wall configurations, presents significant materials and manufacturing challenges. These components are exposed to extreme thermal gradients and mechanical loads, requiring materials that excel in rapid heat transfer from the combustion zone to the coolant while preserving their mechanical properties at high temperatures [1]. To address these design challenges, a dual-material approach has been adopted. Cu-based alloys, selected for their exceptional thermal conductivity, function as thermal liners to effectively manage hot wall temperatures [2]. Ni-based alloys are utilized as high-strength jackets and integrated manifolds, providing structural reinforcement and weight optimization [3]. The Cu-Ni combined structures, offering high thermal conductivity and robust mechanical performance at elevated temperatures, have become a focal point of research and development in the aerospace industry.

To date, various techniques have been developed for producing bimetallic components, including laser-welded sandwich walls, advanced brazing using hot isostatic pressing, and vacuum plasma spray [3]. However, these processes often involve complex methodologies and multiple assembly stages for coolant channel design, resulting in increased production time and costs. Additive manufacturing (AM), particularly directed energy deposition (DED), has emerged as a promising alternative for fabricating metal alloys with complex geometries. DED offers advantages over laser powder bed fusion in terms of build size, speed and can deposit multiple materials on non-planar surfaces.

Previous investigations have explored the challenges of depositing nickel-based superalloys onto copper-based substrates using AM techniques [4–8]. The National Aeronautics and Space Administration (NASA) has successfully employed AM to design a liquid rocket engine, depositing Ni-rich alloy jackets radially on Cu-based liners [2]. Challenges persist in joining Cu- and Ni-based alloys due to their disparate physical and chemical properties. Due to the inherently high thermal conductivity of Cu-based alloys (323 W/m·K), a relatively higher power input is required to compensate for the significant heat dissipation through the C18150 Cu-based alloy at the substrate. Nevertheless, at the upper region of the WAAM-deposited track, localized overheating may occur, which in turn promotes dilution within the top layers [9]. Consequently, the interface experiences a wide composition gradient, promoting defects such as cracks, liquid phase separation, and brittle phase formation. A key challenge in this process is solidification cracking, which arises from the mismatch in solidification behavior between the two alloys.

At NASA, Gradl et al. [10] used electron beam freeform fabrication (EBF3, an electron beam DED technique) to deposit In625 superalloy onto a GRCop-84 copper alloy substrate. They observed significant dilution of the GRCop-84 into the In625 in the first two layers, altering the chemical composition of the Ni-based alloy and resulting in horizontal cracks at the interface between the two materials. Hales et al. [9] employed EBF3 to deposit In625 on GRCop-84 substrates, with the substrates preheated to various temperatures. They observed similar cracking along the boundaries of In625-rich intermediate layers, regardless of the preheating temperature. This cracking was attributed to compositional changes caused by dilution, which lowered the solidus temperature of the adjacent material, increasing the freezing range (i.e. the temperature interval over which the alloy freezes [11]) and the susceptibility to solidification cracking. Preis et al. [12] researched the 15–59 wt.% GRCop-42 mixed with Inconel 625 alloy via the arc melting technique. They found that cracks were introduced during liquid-state processes due to the lack of immiscibility between Cu-depleted and Cu-rich liquid phases in compositions ranging from 30 to 95 wt.% GRCop-42. The study further introduced the CALPHAD method to interpret the results and attributes the failures to three reasons: the formation of cracks and porosity due to Cu-rich liquid entrapment, the phases, and the cracks caused by thermal stress. Rodrigues et al. [13] manufactured the 316 L SS to Inconel 625 functionally graded material using WAAM with direct and smooth-type interfaces, respectively. All samples were found to be well bonded and free of defects, exhibiting superior mechanical properties at the directly transitioned interface.

For directed energy deposition (DED), Kim et al. [14] reported that in 316 L-Inconel 718 functionally graded material (FGM), compositions containing 30 wt.% Inconel 718/70 wt.% SS 316 L and 20 wt.% Inconel 718/80 wt.% SS 316 L tend to form defects due to the presence of ceramic oxides as well as thermal and residual stresses. A local composition detouring method was proposed by Kim et al. [15] to print the defect-free compositionally graded materials with a nonlinear combination of SS316L and Inconel 718. In addition, Liu et al. [16] utilized the DED method to prepare the 316 L and 18Ni300 FGM with a spatially heterogeneous structure, and no obvious defects were found.

The mechanical properties, such as hardness, shear strength, compressive/tensile strength, of the additively manufactured bimetallic joints are also evaluated by many researchers [17–22]. In general, fractures are typically found within the copper alloy regions. The bimetallic interface demonstrates higher strength compared to the copper side, though it remains inferior to the Inconel components. Moreover, the overall thermal conductivity of the structure is enhanced [17–19]. The microstructure of the samples developed by AM methods was also extensively observed by the researchers. The precipitates, such as the Laves phase, is frequently observed at the interface [12,17,18,23].

The above-mentioned studies have provided valuable insights into the challenges of joining Cu- and Ni-based alloys using AM techniques. However, these studies have predominantly relied on experimental approaches to identify cracks and defects, representing a conventional, post hoc strategy in alloy design. There is an urgent need for effective strategies to design crack-free interfaces that can accommodate the dramatic differences in physical properties between these alloys, especially with liquid phase separation during solidification.

To address these gaps, this study employs the ICME (Integrated Computational Materials Engineering) [24,25] approach with supporting experiments. By integrating the CALPHAD method and the solidification cracking susceptibility model – explicitly incorporating phase separation effects not previously considered – we propose an interlayer design with a gradient composition for the C18150-In625 copper and nickel bimetallic system. Experimental validation through WAAM and subsequent heat treatment, coupled with microstructural characterization, facilitates a rigorous assessment of the reliability of the design.

This integrated approach enables the prediction of solidification issues at the design stage, grounded in the underlying physical mechanisms governed by the CSC values. The proposed methodology provides a robust way for designing crack-free bimetallic components, thereby advancing the field of AM for extreme environmental applications.

## 2. Computational modeling and experiments

### 2.1. Cracking susceptibility modeling

Solidification cracking refers to the hot tearing metals undergo during solidification. The crack formation is attributed to stresses resulting from the shrinkage of the formed dendrites and the inability of the remaining liquid to fill back into empty interdendritic spaces. The most important factors that influence the solidification cracking susceptibility are the freezing range and the amount of liquid phase remaining at the last step of solidification [26]. Larger freezing ranges lead to larger weak areas, thus increasing the susceptibility to cracking. Similarly, small amounts of liquid at the end of solidification prevent the filling of the interdendritic spaces, which also promotes cracking defects. It is important to note that the freezing range does not necessarily correlate proportionally with CSC. Solidification cracks can be determined by observing a dendritic fracture surface [27].

In this work, we attempt to design the intermediate layers, comprising a mixture of the parent alloys, to prevent the solidification cracking at the C18150-In625 interface. The simulated interlayer mixtures start from C18150 alloy to In625 superalloy, with a weight percentage step size of 0.05, as presented in Supplementary Table S1. The CSC model developed by Kou [26] considers the lateral growth of two adjacent subgrains during the solidification process. The index reflects both the lateral growth rate of adjacent grains, which promotes bonding and enhances crack resistance, and the length of the grain-boundary liquid channel required to provide sufficient feeding to prevent cracking. A higher index indicates greater crack susceptibility, caused by the slower lateral growth of neighboring grains that delays the bonding needed to resist cracking, and by longer intergranular channels that hinder the liquid feeding required to counteract cracking. Kou’s model is implemented through the Thermo-Calc TC-Python (a Python™ language-based software development kit) interface to estimate the printability, i.e. CSC, as a function of interlayer composition. Such a CSC was calculated with the following formula [28]:

(1) $$\mathrm{CSC}=\max\left|\mathrm{dT}/d\left(\sqrt{f_{s}}\right)\right|,$$

where T represents temperature and *f*_*s*_ denotes the fraction of solid phase during solidification [26]. The Kou model expresses the inverse of the lateral growth rate of dendrites as the solid fraction (*f*_*s*_) approaches unity. Faster lateral growth rates promote rapid dendritic joining, which reduces solidification cracking susceptibility, resulting in a lower CSC value. The solid fraction as a function of temperature was obtained using the Scheil-Gulliver solidification modeling in the Thermo-Calc software with the TCHEA6 database. As the solid fraction data points are not equally spaced, a three-point Lagrange polynomial differentiation was employed to determine its derivative [29]:

(2) $$\left|\frac{dT_{i+1}}{d\left(f_{s}{\;}_{i+1}^{1/2}\right)}\right|\approx \left(\frac{f_{s}{\;}_{i+1}^{1/2}-f_{s}{\;}_{i+2}^{1/2}}{\left(f_{s}{\;}_{i}^{1/2}-f_{s}{\;}_{i+1}^{1/2}\right)\left(f_{s}{\;}_{i}^{1/2}-f_{s}{\;}_{i+2}^{1/2}\right)}\right)T_{i}+\left(\frac{2f_{s}{\;}_{i+1}^{1/2}-f_{s}{\;}_{i}^{1/2}-f_{s}{\;}_{i+2}^{1/2}}{\left(f_{s}{\;}_{i+1}^{1/2}-f_{s}{\;}_{i}^{1/2}\right)\left(f_{s}{\;}_{i+1}^{1/2}-f_{s}{\;}_{i+2}^{1/2}\right)}\right)T_{i+1}+\left(\frac{f_{s}{\;}_{i+1}^{1/2}-f_{s}{\;}_{i}^{1/2}}{\left(f_{s}{\;}_{i+2}^{1/2}-f_{s}{\;}_{i}^{1/2}\right)\left(f_{s}{\;}_{i+2}^{1/2}-f_{s}{\;}_{i+1}^{1/2}\right)}\right)T_{i+2}|$$

Considering the cracking susceptibility increases dramatically at the end of the solidification process, where the remaining liquid is insufficient to backfill the empty interdendritic or intergranular spaces, the CSC was evaluated in the *f*_*s*_ range between 0.9 and 0.98. To assess the printability, the non-equilibrium freezing range and the fraction of undesirable phases were calculated using Scheil-Gulliver and step (phase fraction vs. temperature) diagrams, respectively. High-throughput modeling was carried out using TC-Python to increase the design efficiency for interlayer compositions.

### 2.2. Bimetallic structure fabrication

C18150 and In625 wires with a diameter of 0.045 inches were used for the WAAM process. The chemical compositions of the wires used for printing are listed in Table 1. We leveraged a multi-wire arc AM platform, which allows the deposition rate of each wire to be controlled independently based on the location within the part. Thus, compositional gradients and local variations in alloy content can be generated throughout the printing process.

**Table 1.** A list of the chemical compositions of the wires used in the WAAM printing (wt.%).

Material	Cu	C	Mn	Si	Cr	Mo	Co	Nb	Ti	Al	Fe	Zr	Ni
C18150	98.89	0.00	0.00	0.00	1.00	0.00	0.00	0.00	0.00	0.00	0.00	0.11	0.00
In625	0.00	0.05	0.25	0.25	21.50	9.00	0.50	3.65	0.20	0.20	2.50	0.00	61.90

**Figure 1.**
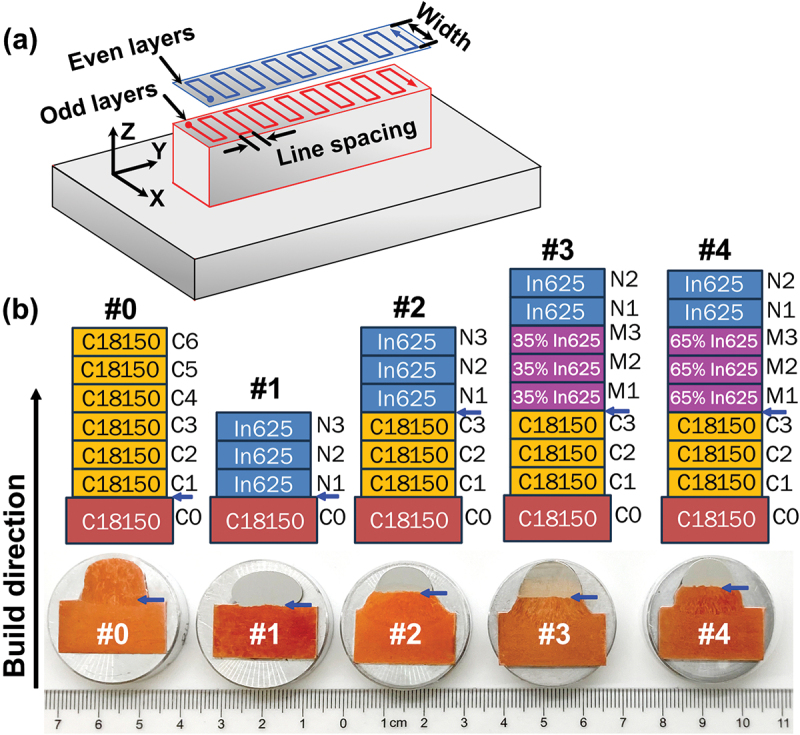
(a) The toolpath used for printing the samples; (b) The stacking of printing layers (top row) and the corresponding overall optical cross-sectional images (bottom row) of the samples. In the top row, C represents C18150 alloy, M represents a mixture of C18150 and In625 alloys, and N represents In625 alloy. The blue arrows mark the area of interest as specific interfaces of each sample for further analysis.

All samples were fabricated using a square raster toolpath featuring a 6 mm width and 3 mm line spacing (Figure 1(a)). While the overall travel direction remained constant, the pattern orientation was reversed for each successive layer. Due to its high thermal conductivity, the C18150 substrate was mounted on an insulating alumina base to enhance preheating. Prior to deposition, the substrate was preheated three times using the arc torch without wire feeding. During printing, the time interval between layers was maintained at less than 60 seconds to preserve heat in the deposit. More detailed information regarding interface printing is available in the Supplementary Materials.

Guided by modeling, five bimetallic specimens are printed on the C18150 substrate with a thickness of 0.5 inches. Figure 1(b) illustrates the stacking of printing layers and the overall optical cross-sectional images of the samples. The layer labels are marked beside each layer, where C represents C18150 Cu alloy, M represents a mixture of C18150 and In625 alloys, and N represents In625 Ni-based superalloy. The detailed printing configuration of the samples is listed in Table 2. Except for sample #0, where C18150 is printed directly onto the C18150 substrate, other samples are printed either with sharp interface transitions (samples #1 and #2) or with a mixture of C18150 and In625 alloys as intermediate layers (samples #3 and #4) between the C18150 substrate and the In625 alloy. Samples #3 and #4 were designed based on cracking susceptibility modeling. In Sample #3, the intermediate layers consist of 35 wt.% In625 and 65 wt.% C18150, whereas in Sample #4, the composition is reversed, with 65 wt.% In625 and 35 wt.% C18150.

**Table 2.** Layers and interface transition type of each WAAM printed sample.

Sample No.	#0	#1	#2	#3	#4
Top layers	6 layers of C18150	3 layers of In625	3 layers of In625	2 layers of In625	2 layers of In625
Intermediate layers	–	–	–	3 layers of 35 wt.% In625	3 layers of 65 wt.% In625
Pre-deposited layers	–	–	3 layers of C18150	3 layers of C18150	3 layers of C18150
Substrate	C18150	C18150	C18150	C18150	C18150
Transition type	No	Sharp	Sharp	Gradual	Gradual

Several studies have investigated the influence of printing sequence on the quality of bimetallic joints [30,31]. Iams et al. [31] reported that printing the GRCop-42 on Alloy 718 resulted in elevated Ni and Fe levels in the GRCop-42 region, leading to the formation of C14 Laves and α(Cr) phases due to significant dilution and convective mixing. While, when the sequence was reversed, a different precipitate, C15_Cr_2_Nb, was observed in the GRCop-42 material. Liu et al. [30] demonstrated that during the deposition of the C18150 on GH4169 alloys, a wide interfacial region would form because of the Marangoni convection and elemental interdiffusion. To improve the interface bonding, Liu suggested using a low thermal conductivity material as a pre-deposition layer or taking some steps to enhance the convection in the melt to widen the interfacial diffusion region. Onuike et al. [18] found that when direct depositing GRCop-84 onto Inconel 718, considerable porosity would occur. This study adopts a deposition sequence commonly employed in liquid rocket engine applications [2], wherein Inconel alloy (In625) is deposited onto a copper substrate (C18150) that has been pre-fabricated by casting or laser powder bed fusion.

### 2.3. Microstructure characterization

Sample slices with 3 mm thickness were prepared by sectioning the WAAM-produced blocks parallel to the build direction. These slices were mechanically polished using an automated polishing and grinding system (E-PREP 4^TM^, Allied High Tech Products, Inc.) with progressively finer grits of sandpapers (*p*-240, *p*-600, *p*-1200, and *p*-2400). Subsequently, they were polished using diamond suspensions of 3, 1, and 0.25 μm diameter, respectively. Finally, the samples were subjected to a vibratory polisher (VibroMet 2 Vibratory Polisher, Buehler Ltd.) with colloidal silica suspension for 10 minutes to achieve scratch-free surfaces.

Microstructural characterization was performed using a scanning electron microscope (SEM, FEI Scios Dual Beam System) in backscattered electron (BSE) mode. Compositional analysis was conducted using energy-dispersive X-ray spectroscopy (EDS, EDAX OctaneElite) equipped on the SEM. Both line and map scans were acquired by the EDS. The width of the line scan was set as 10 μm to get a representative compositional analysis along the build direction. Samples for TEM analysis were extracted from various regions using a focused ion beam (FIB) milling system equipped on the SEM. Phases and precipitates were observed using transmission electron microscopy (TEM, JEOL JEM-2100F).

## 3. Results and discussion

### 3.1. Computational design for crack-free interlayers

**Figure 2.**
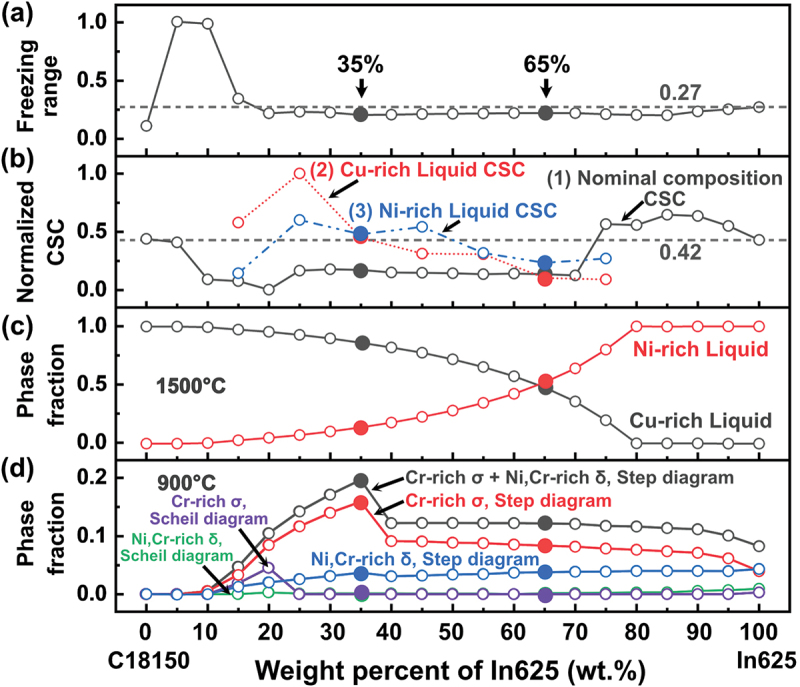
Computed printability of the bimetallic C18150-In625 system. (a) Freezing range, (b) CSC values, (c) Cu- and Ni-rich liquid phase fraction, and (d) fraction of detrimental phases for the mixtures with different weight fractions of In625. The phase fraction in Figure (d) was calculated at 900°C using step diagrams and at the end of solidification using Scheil diagrams.

Figure 2 shows the model prediction results of freezing range, CSC, the fraction of Ni- and Cu-rich liquid phases at 1500°C, and the fraction of undesirable phases at 900°C as a function of the mixing ratio between the C18150 and In625 alloys. The freezing range and CSC values were normalized for practicality. It should be mentioned that 1500°C is chosen considering that it is the temperature at which the alloy systems are entirely liquid, and 900°C is a typical homogenization temperature if any heat treatment is required. As previously mentioned, the compositions of alloy mixtures used in the modeling are listed in Table A1. To evaluate the printability of alloy mixtures, it is necessary to define a maximum value for the freezing range and CSC that helps differentiate between alloys with low and high risk of cracking. Since the parent alloys are printable, the value for the freezing range and the CSC of the parent alloys were considered as benchmark values to compare to estimate the cracking susceptibility. The normalized freezing range value of pure In625 is 0.27 larger than that of pure C18150 (0.11), but the normalized CSC for both parent alloys is similar, close to 0.42. It is important to note that we focus on the relative value difference rather than discussing the absolute values. Apparently, if one uses the freezing range value to guide the design, a wide composition range of alloy mixture between C18150 and In625 (i.e. from ~20 to 100 wt.% In625) will be considered as suitable for printing. Instead, if we consider using the normalized CSC value to guide the design, the computed CSC based on the nominal alloy mixture composition shown in Figure 2(b) predicts the feasible range for printing from ~5 to ~70 wt.% In625. Combining these two design factors, the alloy mixtures containing In625 between 20 and 70 wt.% are found to be possible for printing.

However, as indicated in Figure 2(c), when In625 content is in the range of 10 wt.% to 80 wt.%, liquid phase separation could occur, leading to the formation of Cu- and Ni-rich liquids. This is attributed to the miscibility gap of both liquid and fcc phases originating from the Cu-Cr-Ni ternary system. Therefore, the liquid phase separation results in two distinct solidification paths. Considering this, we display two additional CSC curves in Figure 2(b), predicted using Ni-rich and Cu-rich liquid composition at the onset of the liquid phase separation.

During solidification, the presence of two liquids could generate considerable residual stress due to the difference in freezing ranges and mismatches in the coefficient of thermal expansion of both compositions. Moreover, after the solidification is finished, a high fraction of brittle phases, such as δ and σ, is predicted, as illustrated in Figure 2(d), which could also affect the printability of the bimetallic system. It should be noted that the Scheil non-equilibrium model may not predict any noticeable formation of detrimental phases. Therefore, to adopt a more cautious approach, the fraction of undesirable phases was estimated using equilibrium diagrams at 900°C. In this study, it is assumed that below 900°C, phase transformations are kinetically frozen during the manufacturing process.

Based on the above computational modeling, in this work, we proposed two graded alloy samples by introducing an intermediate composition block. One is sample #3 with 35 wt.% In625 and the other one is sample #4 with 65 wt.% In625.

It should be noted that sample #3 shows the computed CSC value as low as that of sample #4 when using the nominal alloy mixture composition as the input. However, if the liquid phase separation should be considered in the CSC prediction by taking into account the liquid phase composition, sample #3 will be more prone to the solidification crack due to the higher CSC values illustrated in Figure 2(b). The samples with no intermediate layers (samples #0, #1, and #2) are also printed as reference samples.

### 3.2. Solidification cracking susceptibility of interlayers

**Figure 3.**
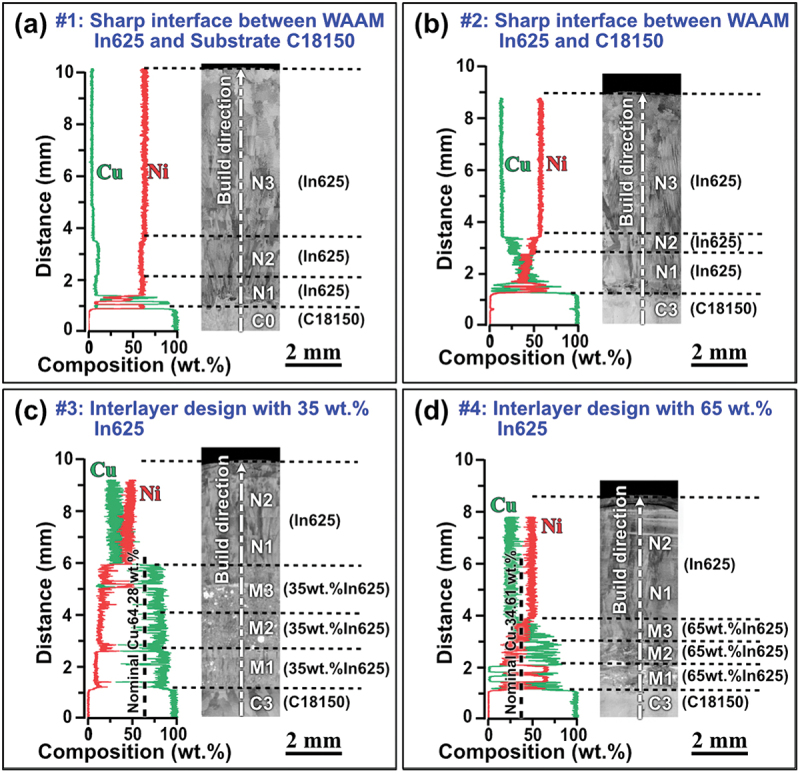
The EDS line scan along build direction and BSE images of cross–sections of samples (a) #1, 3-layer In625 directly depositing on the C18150 substrate, (b) #2, 3–layer In625 depositing on 3–layer C18150, (c) #3, 2–layer In625 depositing on 3–layer C18150 with 3– layer 35 wt.% In625 mixture interlayer in between, and (d) #4, 2–layer In625 depositing on 3– layer C18150 with 3–layer 65 wt.% In625 mixture interlayer in between. In (b)–(d), the first two layers C1 and C2, as well as the substrate layer C0 are omitted for clarity.

To comprehensively understand the microstructure and its relation to the interlayer design, we observed the interface between In625 and C18150 for samples #1 and #2, and between the interlayers and the pre-deposited C18150 layers for samples #3 and #4 (as indicated by blue arrows in Figure 1(b)). The EDS and BSE observations on the cross-sections of samples #1 to #4 are shown in Figure 3. The composition variation along the White dashed arrow lines parallel with the build direction is illustrated for each sample. The layer labels and interfaces are marked in the BSE micrographs. It can be observed that all the samples have variations in chemical composition from the interface of N1/C0, N1/C3, or M1/C3. The content of Cu decreases from the bottom C18150 side to the top In625 side. Meanwhile, the content of Ni is going in the opposite trend. Interestingly, many White blocks are found in the interlayers of both samples #3 and #4. There are no large pores or voids found in Figure 3. For reference, the microstructure of sample #0, consisting of pure C18150 throughout the substrate and printed layers, is presented in the Supplementary Materials. The absence of visible cracks at different length scales in sample #0 confirmed the feasibility of printing C18150 alloy onto the C18150 substrate, demonstrating successful control of heat input during the process. In this section, we will focus on cracking formation and precipitates, and phase separation will be discussed in the next section.

**Figure 4.**
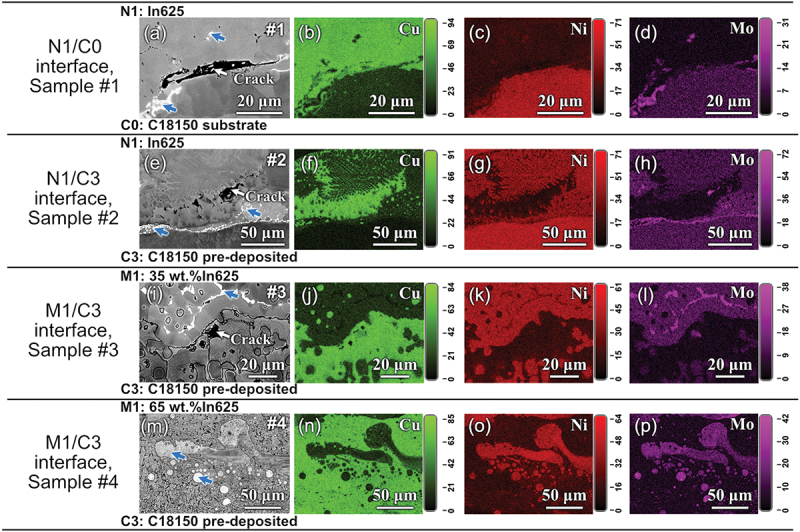
BSE and EDS images showing the cracks region of (a–d) N1/C0 boundary of sample #1, (e–h) N1/C3 boundary of sample #2, (i–l) M1/C3 boundary of sample #3, and (m-p) M1/C3 boundary of sample #4. The colorful images are the element distribution maps obtained by EDS. White arrows represent the cracks, and blue arrows indicate the σ phase.

Figure 4 presents representative BSE and EDS images of the areas near the layer boundaries of interest, where White arrows indicate cracks. Generally, most cracks are found in the separate Cu-rich FCC phase, which solidifies later in the printing process. The images of sample #0 (pure C18150) are not displayed because no cracks were found in it. We observed the cracks in the whole cross-sectional areas of all the #1-#4 samples. The statistical crack density is calculated as the total length of the cracks divided by the area of the sample cross-section. A summary of the cracks in all the samples is presented in Table 3, which shows that the crack density decreases in the order #1 > #2 > #3 > #4.

**Table 3.** A summary of crack analysis in different samples.

	#1	#2	#3	#4
Main Location	At Ni-/Cu-rich FCC boundaries and in Cu-rich FCC	In Cu-rich FCC	In Cu-rich FCC	–
Length density (10^−5^ μm/µm^2^)	4.05	1.42	1.12	0
Average length (μm)	39.7 ± 31.8	18.4 ± 10.3	28.5 ± 14.5	0

In sample #1, with a sharp transition from C18150 to In625 and no pre-deposited C18150 layers, cracks are found both on the Cu-/Ni-rich FCC phase boundaries and in the Cu-rich FCC phases. An example of a crack formed on the phase boundary is illustrated by the White arrow in Figure 4(a). The cooling rate of the initial few layers is typically very high [32], especially when printing on the copper alloy, due to its high thermal conductivity. Therefore, the In625 superalloy in sample #1, with no pre-deposited C18150 layers, experienced the highest cooling rate. Such rapid cooling leads to steep temperature gradients, causing differential thermal expansion and contraction. As a result, residual stress and crack density increase [33]. Sample #2 also has a sharp transition from C18150 to In625 alloy. While with three pre-deposited C18150 layers, the cracks in sample #2 (as shown in Figure 4(e)) are smaller and fewer than those in sample #1. This means adding a three-layer deposit of C18150 can increase the temperature of the substrate, therefore significantly reducing the crack density, as in sample #2.

After introducing a 35 wt.% In625 intermediate layer, the cracks observed in sample #3 are reduced. The defects exhibit an average size of approximately 28.54 μm, as shown in Figure 4(i). In addition, cracks were eliminated in sample #4, designed with the 65 wt.% In625 interlayers. These results suggest that intermediate layers effectively reduce or eliminate crack formation.

According to Figure 2(b), the calculated CSC values for both samples #3 and #4 by considering the composition of the alloy mixture as the input are close to each other and are much less than the normalized CSC value (0.42) of the parent alloys. This means that both alloys are not prone to cracking. However, the micrographs in Figure 4(i) show cracks in the 35%In625 interlayer of sample #3. So, the CSC values of the separated liquid phases before solidification were further checked to explore the crack formation mechanisms in more detail for these two samples.

**Figure 5.**
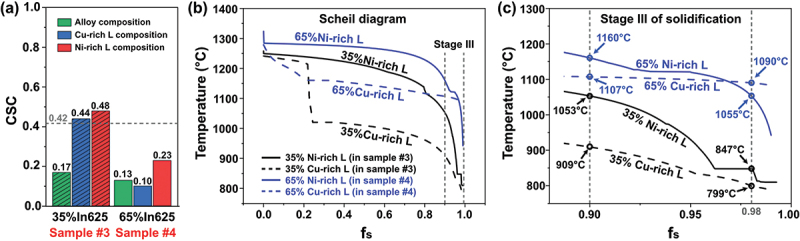
Effect of liquid phase separation on printability: (a) CSC of the nominal alloy mixtures containing 35 and 65 wt.% In625, and CSC of the Cu- and Ni-rich liquid resulting from the liquid phase separation of each alloy mixture, (b) Scheil curves obtained by using the composition of the Cu- and Ni-rich liquids of the 35%In625 and 65%In625 alloys, and (c) a magnification of stage III of solidification (0.9 < f_s_ < 0.99) in (b).

Figure 5(a) shows the CSC values of the intermediate layers of samples #3 (35%In625) and #4 (65%In625) in two different cases. In the first case, the CSC is calculated using the nominal alloy mixture composition for the specific interlayer design (green bars). This results in CSC values of 0.17 and 0.13 for the interlayers with 35 and 65 wt.% In625, respectively. These values are also plotted in Figure 2(b).

However, considering liquid phase separation in both interlayer designs, the second case predicts CSC using two liquid phase compositions at the onset of solidification. The corresponding compositions are listed in Table 4. Surprisingly, for sample #3 (35%In625), much higher CSC values are obtained. Using the Cu-rich liquid phase composition at the onset of solidification, the CSC is 0.44 (blue bar for sample #3 in Figure 5(a)), and for the Ni-rich liquid phase composition, it is 0.48 (red bar for sample #3 in Figure 5(a)). Both values exceed the maximum set value (0.42), the normalized CSC for both parent alloys, indicating that liquid separation in sample #3 can lead to a higher propensity for crack formation.

**Table 4.** Chemical compositions (wt.%) of the characteristic liquid state of the 35%In625 and 65%In625 alloys. These compositions were calculated at the liquidus temperature and are used to estimate the csc in Figures 2 and 5.

Sample ID	Composition, wt.%												
	Cu	Ni	Cr	Zr	Mo	Nb	C	Al	Ti	Fe	Co	Si	Mn
35%ln625	64.28	21.67	8.18	0.07	3.15	1.28	0.02	0.07	0.07	0.88	0.18	0.09	0.09
35%Cu-rich L	78.70	9.80	4.50	0.00	0.10	0.00	0.00	0.10	0.00	0.50	0.10	6.10	0.10
35%Ni-rich L	6.10	45.70	22.90	0.30	15.30	6.30	0.10	0.00	0.20	2.20	0.50	0.40	0.00
65%ln625	34.61	40.24	14.33	0.04	5.85	2.37	0.03	0.13	0.13	1.63	0.33	0.16	0.16
65%Cu-rich L	65.50	26.00	6.50	0.00	0.20	0.10	0.00	0.30	0.00	0.90	0.20	0.00	0.30
65%Ni-rich L	10.90	51.00	20.30	0.10	10.20	4.10	0.10	0.00	0.20	2.20	0.50	0.30	0.10

Interestingly, using the same CSC estimation approach, both Cu-rich and Ni-rich liquid phases do not introduce much higher CSC values for sample #4 (65%In625), as shown in Figure 5(a). One is 0.10 for Cu-rich liquid, and the other is 0.23 for Ni-rich liquid.

Considering liquid phase separation, the Ni- and Cu-rich liquids in the 35%In625 interlayer of sample #3 have higher cracking susceptibility than the liquids in the 65%In625 layer of sample #4. These CSC calculations, based on liquid phase composition rather than alloy composition for the interlayer, are consistent with experimental observations discussed earlier and shown in Figure 5.

Hot cracking has been reported when mixing Cu and Ni alloys [9]. However, the failure mechanism is still a matter of debate. Possible mechanisms include solidification cracking and liquation cracking. Furthermore, as liquid phase separation is exhibited in these bimetallic systems, cracking associated with the shrinkage of the Cu-rich islands must be considered [12]. Defining the failure mechanism is a complex task, as identifying the exact moment of crack formation is difficult. Furthermore, multiple failure mechanisms may simultaneously contribute to crack formation. In this work, we focus on evaluating solidification cracking with consideration of liquid phase separation.

**Figure 6.**
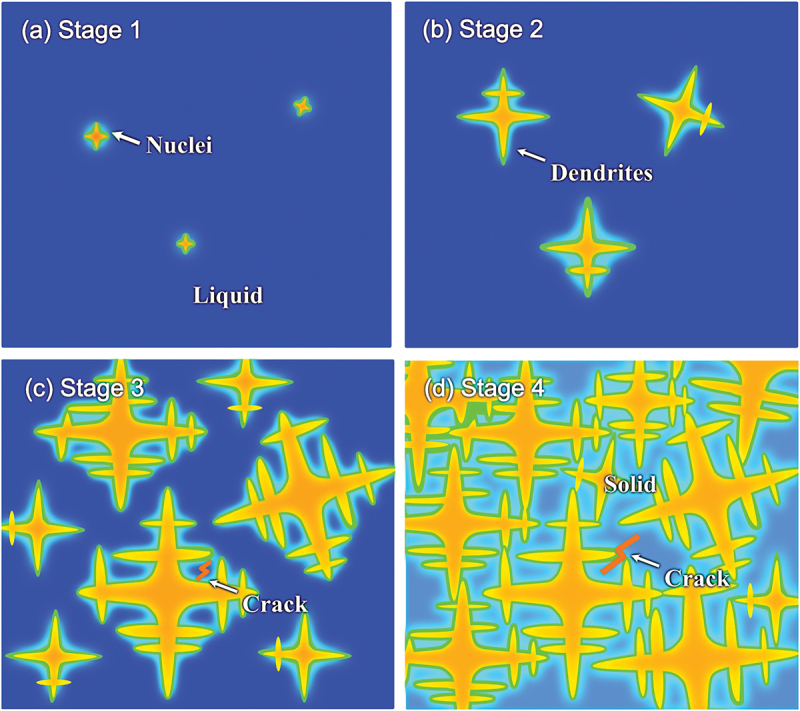
Illustration of the solidification process: (a) nuclei begin to form (stage 1), (b) nuclei grow without disrupting the flow of the liquid phases (stage 2), (c) complex interdendritic tunnels developed and preventing the free movement of the remaining liquid result in high cracking susceptibility (stage 3), and (d) the solidification process finishes (stage 4).

According to Borland [34,35], the solidification process can be divided into four stages as seen in Figure 6: (1) At the onset of solidification, the solid nuclei (the formation of the first solid phase) begin to form homogeneously and heterogeneously; (2) As the temperature decreases, the nuclei grow without disrupting the flow of the liquid phases; (3) As the solid fraction increases to the range above ~0.9 [28], complex interdendritic networks form, creating interconnected tunnels that become progressively filled with the constrained liquid phase. This microstructural evolution significantly impedes the mobility of the remaining liquid phase, ultimately leading to restricted mass transport during the final stages of solidification. The liquid-solid phase formation, shrinkage, and thermal stress generated during the fast cooling may lead to crack formation in the copper-rich liquid region [36]. At this stage, cracking susceptibility reaches its peak; (4) Finally, the solidification process finishes. Generally, to minimize cracking susceptibility, it is advisable to maintain the third stage of solidification at the lowest possible temperature range [28].

It is necessary to clarify that the physical model presented here is intended to provide a general conceptual framework for the design approach, rather than to capture every detail of the complex AM environment. Accurately representing all such details would require comprehensive multi-physics simulations, which are beyond the current scope of this work.

Figure 5(b,c) show the Scheil curves of the liquid phases in the interlayer with 35 and 65 wt.% In625 (noted as 35In625 and 65In625, respectively). As seen in Figure 5(c), the 65Cu-rich L in sample #4 crosses the third stage of solidification in a short temperature range of 17°C (the difference between the beginning, 1107°C, and the end, 1090ºC), which decreases its tendency to crack. On the contrary, the 35Cu-rich L of sample #3 undergoes the third stage in a temperature range of 110°C, which may lead to crack formation. The smaller temperature variation during the third stage of solidification contributes to the fabrication of the crack-free sample (Figure 5(c)). In addition, the cracks at the interface between the Ni- and Cu-rich islands, as well as those within the Cu-rich regions, can be attributed to the different solidification temperature ranges of the Cu- and Ni-rich liquids. As shown in Figure 5(c), the Cu-rich liquid in the 35% In625 sample solidifies after the Ni-rich liquid, leading to the confinement of the remaining Cu-rich liquid by the solidified Ni-rich matrix. This restriction generates tensile stresses due to the limited contraction of the Cu-rich liquid, thereby promoting crack formation.

During the printing process from Cu alloy to Ni superalloy, solidification cracking significantly contributes to the cracking of bimetallic components. Therefore, cracks can be eliminated by minimizing the solidification cracking susceptibility of the alloy. However, due to the liquid phase separation observed at the bimetallic interface, the solidification cracking susceptibility, i.e. CSC, of each liquid phase must be considered, rather than assuming it is identical to the original high-temperature liquid phase of the alloy composition.

In conclusion, the results suggest that in the case of liquid phase separation, it is advisable to consider the cracking susceptibility of each liquid rather than the entire alloy. Based on this, we successfully designed crack-free intermediate layers for bimetallic sample #4 with low CSC values in the liquid phases during solidification. However, further investigation is needed to address additional challenges, including elemental dilution, phase separation, and the formation of detrimental precipitates.

### 3.3. Interlayer microstructure and phase stability

#### 3.3.1. Dilution and chemical transition along the build direction

Different materials exhibit varying dilution rates during printing deposition, leading to the mixing of subsequent layers with portions of the preceding layers [36]. This mixing can result in chemical inaccuracies and compromised performance. As reported in [37], the dilution rate is directly proportional to the melt pool size. In our experiments, the melt pool width exceeded 3 mm, indicating a significant potential for dilution. The EDS results along the build direction in Figure 3 show a significant amount of Cu in the In625 layers of all the samples. This indicates the dilution occurred in these samples, i.e. the freshly deposited layer partially remelted the previous layer and led to the mixing of elements, e.g. Cu, from the previous layer into it. Figure 3(a) shows that sample #1 exhibited a relatively sharp boundary between the N1 (In625) and C0 (C18150) layers. In contrast, the incorporation of three pre-deposited C18150 layers in sample #2 significantly improved the interfacial integrity, primarily due to the elevated substrate temperature and lower cooling rate compared to sample #1. Moreover, in sample #2, the Cu dilution is stronger than that in sample #1. This may be due to the pre-deposited heating up the substrate and providing more energy for the subsequent element dilution.

Mutual solubility dilution occurs through the C18150 substrate, the interlayer with alloy mixture, and the final In625 layers. It can be seen that the dilution makes the copper concentration higher than the nominal Cu concentration of the interlayers for samples #3 (64.28 wt.%) and #4 (34.61 wt.%), as shown by the vertical dashed lines in the EDS results in Figure 3(c,d). This high Cu concentration will undoubtedly impact the formation of cracks in the material due to changes in the CSC value of the Cu-rich liquid. Thus, dilution should be carefully considered in developing a more precise design model for the printing of functionally graded materials, particularly when utilizing the WAAM technique.

#### 3.3.2. Phase separation

**Figure 7.**
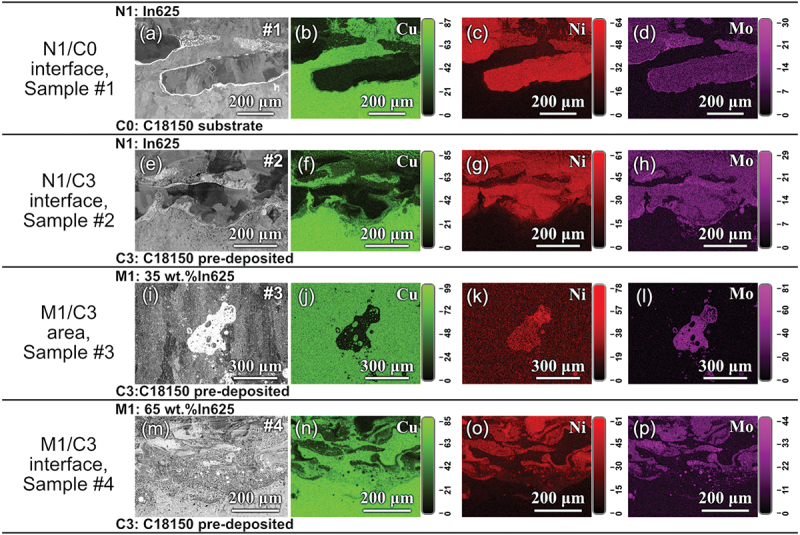
BSE and EDS maps showing the phase separation in localized regions for different samples: (a–d) N1/C0 boundary of sample #1, (e–h) N1/C3 boundary of sample #2, (i–l) M1/C3 area of sample #3, and (m–p) M1/C3 boundary of sample #4. The colorful images shown in the 2nd, 3rd, and 4th columns are EDS element distribution maps for Cu, Ni, and Mo, respectively.

Significant phase separation was observed in all the samples, as illustrated in Figure 7. For samples #1 and #2, phase separation was primarily observed near the first interface between the C18150 and In625 alloys (i.e. N1/C0 interface for #1 and N1/C3 interface for #2), as shown in the micrographs in Figure 7(a–h). EDS maps confirmed that this phase separation primarily involved Cu-rich face-centered cubic (FCC) and Ni-rich FCC phases. In contrast, for samples #3 and #4, phase separation occurred not only along the M1/C3 boundaries (Figure. 7(i–p)) but also throughout the intermediate layers (Figure. 3(c,d)). This resulted in the formation of large-sized, segregated Ni- and Cu-rich regions within the three intermediate layers. The BSE images in Figure. 7(i,m) reveal distinct, bright White Ni-rich regions (referred to as Ni-rich islands hereafter) exhibiting irregular or spherical shapes. As further analyzed in a later section, these Ni-rich islands consist of a Ni-rich FCC phase embedded with high-density fragmental σ phases.

Based on the computed phase fraction in Figure 2(c), we can calculate the RNC, i.e. the Ratio of Ni-rich liquid to Cu-rich liquid phases. Sample #3 (35 wt.% of In625 superalloy) has a lower RNC (~0.148). In comparison, sample #4 (65 wt.% of In625 superalloy) has a higher RNC (~1.106), which caused the formation of many large bright White Ni-rich islands in the intermediate layers. We also statistically analyzed the Ni-rich islands with an equivalent diameter larger than 50 μm from BSE images in Figure. 3(c,d). The area fraction of Ni-rich islands, *E*, is calculated for samples #3 and #4 using the following equation:

(3) $$E=A_{i}/A_{t}$$

where $A_{i}$ is the sum area of the bright White Ni-rich islands, which contain the σ or δ phases, and $A_{t}$ is the cross-sectional area of all intermediate layers. The *E* values are 8.35% and 17% for samples #3 and #4, respectively. Both the *RNC* and *E* values imply that sample #4 has stronger phase separation than that of sample #3. More separated phase islands in sample #4 lead to a much higher fluctuation of the composition curve than that of sample #3, as shown in Figure 3.

#### 3.3.3. Microstructure analysis of intermetallic phases

Small-sized σ phases were observed in all samples alongside Cu-rich FCC, Ni-rich FCC, and BCC (Cr) phases. For samples #1 and #2, these precipitates primarily formed at the interfaces of the Ni-rich and Cu-rich regions. The sharp transition at the interface led to the formation of granular or fragmental σ phases in the narrow Cu-rich and Ni-rich FCC phase boundaries, as indicated by the blue arrows in Figure 4(a,e), respectively.

**Figure 8.**
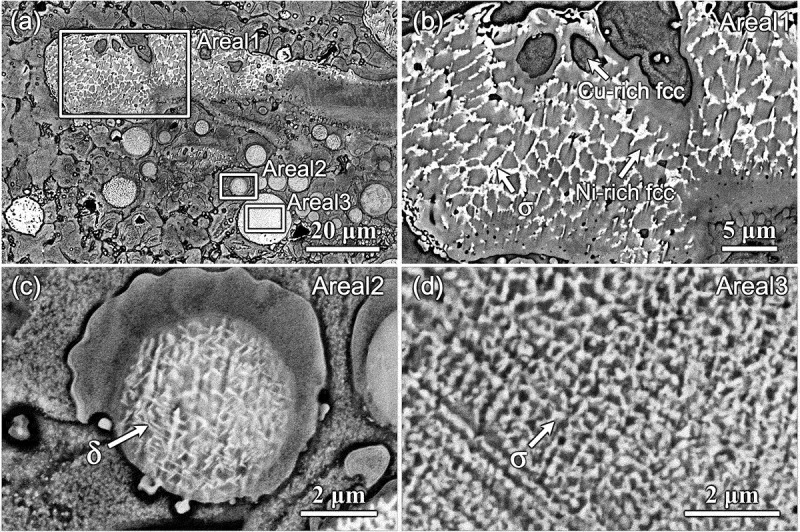
The observation of the bright White islands in sample #4 near the M1/C3 boundary: (a) a region with irregular and spherical shaped islands, (b–d) magnified observations of Areas 1, 2, and 3, respectively.

In addition to the σ phase, the δ phase was observed in samples #3 and #4, predominantly within the bright White Ni-rich islands. The precipitate distribution in sample #3 was similar to that observed in sample #4. Figure 8 presents a detailed observation of the phases found within the Ni-rich islands of sample #4. Figure 8(a) illustrates the diverse shapes of these islands, which are enriched in Ni, Mo, Nb, and Cr. These elements primarily originate from the In625 superalloy, with minor diffusion of Cu from the C18150 alloy during the deposition. Figure 8(b–d) provide magnified views of the precipitates in Areas 1–3 within Figure 8(a). Observations revealed numerous small, irregularly shaped σ phases in Areas 1 and 3, and a high density of δ phases in Area 2. The chemical composition of the three areas and σ phases is listed in Table 5.

**Table 5.** The composition (wt.%) of the three areas and the σ phase in Figure 8.

wt.%	Al	Si	Ti	Cr	Mn	Fe	Co	Ni	Cu	Zr	Nb	Mo
Area1	0.27	0.22	0.09	19.41	0.05	0.18	0.00	45.73	14.65	0.10	4.13	15.17
Area2	0.17	0.22	0.12	19.77	0.06	0.24	0.02	44.98	16.32	0.00	3.89	13.90
Area3	0.36	0.65	0.27	19.84	0.43	0.44	0.20	42.01	11.44	0.24	4.74	19.74
σ	0.33	0.90	0.00	26.21	0.00	0.17	0.00	29.68	2.39	0.41	4.47	35.96

**Figure 9.**
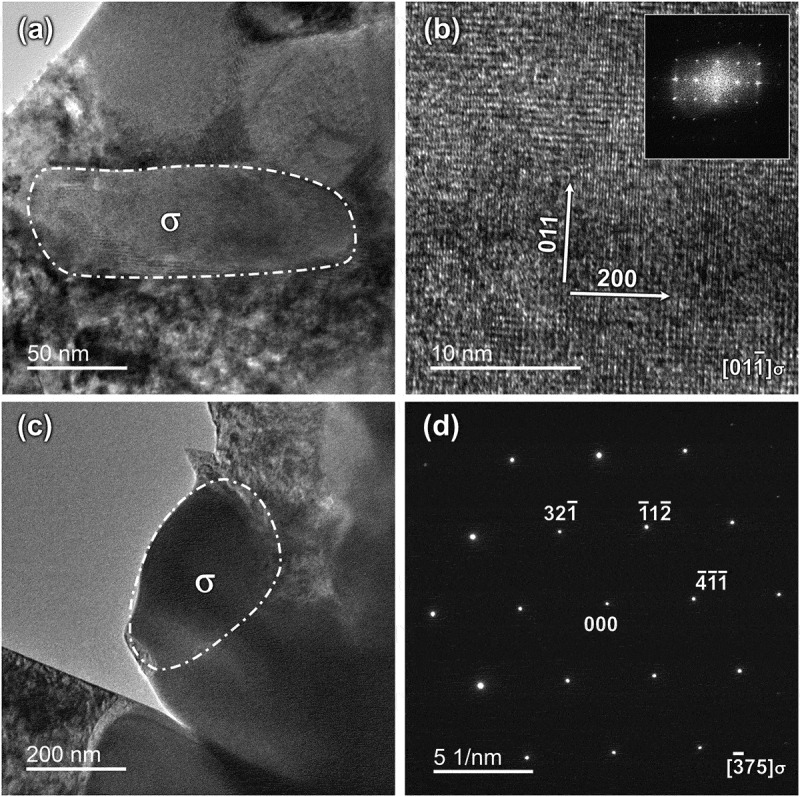
TEM micrographs of the as-built sample #4. (a) and (c) The transmission images of Areas 1 and 3 in Figure 8, respectively. (b) The high-resolution image of the marked precipitate in (a). (d) The selected area diffraction patterns of the marked precipitate in (c).

TEM samples were prepared by FIB from the exact locations of Areas 1 and 3, and their observations are presented in Figure 9. The TEM results confirmed that the tiny particles in both areas are the tetragonal σ phase, with a space group of P4_2_/mnm [38,39]. The σ phase in Areas 1 and 3 exhibits zone axes of [01$\overset{ˉ}{1}$] and [$\overset{ˉ}{3}$75], respectively.

### 3.4. Phase stability study of intermetallic compounds after long-term heat treatment

While introducing mixed C18150-In625 alloy interlayers effectively reduces or eliminates crack formation, it also causes significant phase separation and detrimental phase formation. Moreover, phase separation and detrimental phases can lead to mechanical failure during subsequent service [38]. Preis et al. [12] also confirmed that the precipitate phases are potentially leading to the cracking. High-temperature homogenization typically dissolves undesired phases. Therefore, we employed long-term heat treatments at elevated temperatures to evaluate the phase stability within the microstructure of the as-built samples. It is worth mentioning that, since the focus of this paper is on using computational calculation methods to predict and design crack-free bimetallic samples, we intentionally refrain from going too far beyond that scope. Thus, this study only discusses the influence of heat treatment and does not include any thermal or mechanical property testing.

**Figure 10.**
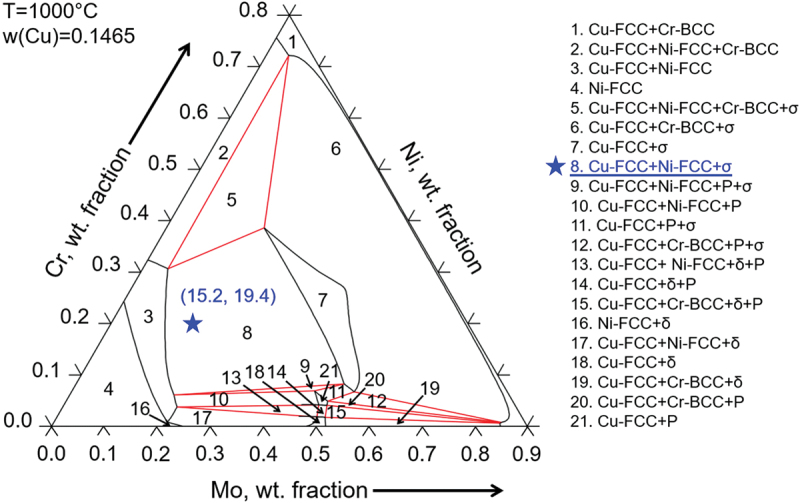
Calculated isothermal section of the Ni–Cr–Mo phase diagram at 1000 °C.

Before conducting experiments, phase diagram calculations can provide useful insights into the formation of these intermetallics. For example, Figure 10 is the isothermal section of the Ni-Cr-Mo-Cu phase diagram under 1000°C at a constant Cu content of 14.65 wt.%, which is based on the composition of Area 1 shown in Figure 8. It shows that the σ phase forms simultaneously with the Cu- and Ni-rich FCC phases, as highlighted by the blue star in Figure 10, which shows a similar content with 15.2 wt.% Mo and 19.4 wt.% Cr as Area 1. This also indicates that an isothermal heat treatment at such a high temperature is not able to successfully dissolve these detrimental intermetallic phases.

**Figure 11.**
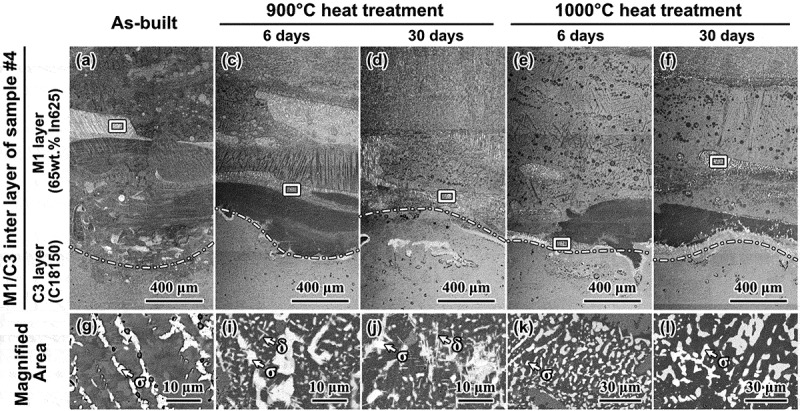
The microstructure of the as-built and heat-treated sample #4: (a) the as-built specimen, (b) and (c) the specimens heat-treated under 900° C for 6 days and 30 days, respectively, (e) and (f) the specimens heat-treated under 1000°C for 6 days and 30 days, respectively. Images (g–l) are magnified observations corresponding to images (a–f).

Besides the phase diagram calculation, slices from sample #4 were heat treated with argon protection at 900°C and 1000°C (close to the melting point of the copper alloy) for 6 and 30 days, respectively, to experimentally evaluate the phase stability of unwanted intermetallic precipitates. Figure 11 shows the BSE images of the as-built and heat-treated conditions of sample #4 near the M1/C3 boundaries, specifically at the interface between 65 wt.% In625 and the 100% C18150 layers (see Figure 1(b)). The results indicate that phase separation persisted even after prolonged heat treatment at elevated temperatures. Furthermore, the precipitates near the M1/C3 boundary increased in size and density after long-term homogenization at both temperatures. Therefore, both the calculation and experimental results show that the phase separation and detrimental phases cannot be resolved by heat treatment alone. Instead, new methods need to be identified.

Recent studies have investigated the incorporation of a third alloy between Ni-based and Cu-based alloys [23,40]. For example, Preis et al. [40] employed IN718 and GRCop-42, with pure Ni serving as the intermediate alloy. Although this approach resulted in less severe phase separation (liquid miscibility), it did not entirely eliminate the formation of detrimental phases. Given the limitations of heat treatment in resolving phase separation and detrimental phase formation, alternative approaches should be considered. (1) Optimized Intermediate Layer Materials: By introducing intermediate layer materials that exhibit enhanced compatibility, it becomes possible to suppress liquid phase separation and mitigate the formation of harmful secondary phases. To facilitate such advancements, the CALPHAD-based ICME design framework developed in this work will assist in systematically evaluating commercially available candidates for use as intermediate layers. This framework will be made available to support targeted material design efforts. (2) Advanced Processing Techniques: Employing other advanced processing techniques as a hybrid manufacturing approach, such as thermal/cold spray, to bypass the temperature range of the detrimental phase formation, potentially reducing phase separation. And this also requires a careful design based on the phase-level research toolkits such as CALPHAD.

## 4. Conclusions

This study aimed to understand phase behavior during microstructure evolution in the WAAM process and guide the design of crack-free intermediate layers between C18150 and In625 alloys using a CALPHAD-based ICME framework.

- The Kou model of solidification CSC, based on the CALPHAD database, successfully predicted the printability of alloy mixtures. Introducing an interlayer with 65 wt.% In625 between parent alloys effectively prevented crack formation, even in the presence of unavoidable liquid phase separation.
- A quantitative correlation between liquid phase separation and cracking susceptibility was established. For a reliable prediction of solidification CSC, it is essential to focus on the individual separated liquid phase rather than the nominal composition of the alloy. This was confirmed through experiments and design on the interlayer composition with 35 vs. 65 wt.% In625.
- Phase separation and detrimental σ and δ phases occurred in the intermediate layers of the samples, which could not be eliminated by long-term (up to 30 days) heat treatment at elevated temperatures (900–1000ºC). The undesirable phases that have grown larger may further degrade the thermal and mechanical performance, which requires more attention before it can be applied for usage.

## Supplementary Material

Supplemental Material
