# Supplementary material for: CALPHAD-guided interlayer design for crack-free additive manufacturing of copper C18150 – Inconel 625 bimetallic structures
Source: Sci Technol Adv Mater. 2025 Nov 26;26(1):2587393. doi: 10.1080/14686996.2025.2587393 (PMC12667290; doi:10.1080/14686996.2025.2587393)
Supplement: Supplemental Material [file TSTA_A_2587393_SM9648.docx]

# **Supplementary Materials**

Below is a table showing microstructure of sample #0, which has pure C18150 throughout the substrate to the printed layers.

***Table S1.*** Chemical composition (wt.%) of the mixtures between C18150 and In625.

| ID | In625 fraction | Cu | Ni | Cr | Zr | Mo | Nb | C | Al | Ti | Fe | Co | Si | Mn |
| --- | --- | --- | --- | --- | --- | --- | --- | --- | --- | --- | --- | --- | --- | --- |
| C18150 | 0.00 | 98.89 | 0.00 | 1.00 | 0.11 | 0.00 | 0.00 | 0.00 | 0.00 | 0.00 | 0.00 | 0.00 | 0.00 | 0.00 |
| 0.05ln625 | 0.05 | 93.95 | 3.10 | 2.03 | 0.10 | 0.45 | 0.18 | 0.00 | 0.01 | 0.01 | 0.13 | 0.03 | 0.01 | 0.01 |
| 0.10In625 | 0.10 | 89.00 | 6.19 | 3.05 | 0.10 | 0.90 | 0.37 | 0.01 | 0.02 | 0.02 | 0.25 | 0.05 | 0.03 | 0.03 |
| 0.15ln625 | 0.15 | 84.06 | 9.28 | 4.08 | 0.09 | 1.35 | 0.55 | 0.01 | 0.03 | 0.03 | 0.38 | 0.08 | 0.04 | 0.04 |
| 0.20ln625 | 0.20 | 79.11 | 12.38 | 5.10 | 0.09 | 1.80 | 0.73 | 0.01 | 0.04 | 0.04 | 0.50 | 0.10 | 0.05 | 0.05 |
| 0.25n625 | 0.25 | 74.17 | 15.48 | 6.13 | 0.08 | 2.25 | 0.91 | 0.01 | 0.05 | 0.05 | 0.63 | 0.13 | 0.06 | 0.06 |
| 0.30ln625 | 0.30 | 69.22 | 18.57 | 7.15 | 0.08 | 2.70 | 1.10 | 0.02 | 0.06 | 0.06 | 0.75 | 0.15 | 0.08 | 0.08 |
| 0.35ln625 | 0.35 | 64.28 | 21.66 | 8.18 | 0.07 | 3.15 | 1.28 | 0.02 | 0.07 | 0.07 | 0.88 | 0.18 | 0.09 | 0.09 |
| 0.40In625 | 0.40 | 59.33 | 24.76 | 9.20 | 0.07 | 3.60 | 1.46 | 0.02 | 0.08 | 0.08 | 1.00 | 0.20 | 0.10 | 0.10 |
| 0.45ln625 | 0.45 | 54.39 | 27.86 | 10.23 | 0.06 | 4.05 | 1.64 | 0.02 | 0.09 | 0.09 | 1.13 | 0.23 | 0.11 | 0.11 |
| 0.50ln625 | 0.50 | 49.45 | 30.95 | 11.25 | 0.06 | 4.50 | 1.83 | 0.03 | 0.10 | 0.10 | 1.25 | 0.25 | 0.13 | 0.13 |
| 0.55In625 | 0.55 | 44.50 | 34.04 | 12.28 | 0.05 | 4.95 | 2.01 | 0.03 | 0.11 | 0.11 | 1.38 | 0.28 | 0.14 | 0.14 |
| 0.60ln625 | 0.60 | 40.55 | 36.52 | 13.10 | 0.05 | 5.31 | 2.15 | 0.03 | 0.12 | 0.12 | 1.48 | 0.30 | 0.15 | 0.15 |
| 0.65ln625 | 0.65 | 34.61 | 40.24 | 14.33 | 0.04 | 5.85 | 2.37 | 0.03 | 0.13 | 0.13 | 1.63 | 0.33 | 0.16 | 0.16 |
| 0.70In625 | 0.70 | 29.67 | 43.33 | 15.35 | 0.03 | 6.30 | 2.56 | 0.04 | 0.14 | 0.14 | 1.75 | 0.35 | 0.18 | 0.18 |
| 0.75ln625 | 0.75 | 24.72 | 46.42 | 16.38 | 0.03 | 6.75 | 2.74 | 0.04 | 0.15 | 0.15 | 1.88 | 0.38 | 0.19 | 0.19 |
| 0.80ln625 | 0.80 | 19.78 | 49.52 | 17.40 | 0.02 | 7.20 | 2.92 | 0.04 | 0.16 | 0.16 | 2.00 | 0.40 | 0.20 | 0.20 |
| 0.85In625 | 0.85 | 14.83 | 52.62 | 18.43 | 0.02 | 7.65 | 3.10 | 0.04 | 0.17 | 0.17 | 2.13 | 0.43 | 0.21 | 0.21 |
| 0.90ln625 | 0.90 | 9.89 | 55.71 | 19.45 | 0.01 | 8.10 | 3.29 | 0.05 | 0.18 | 0.18 | 2.25 | 0.45 | 0.23 | 0.23 |
| 0.95ln625 | 0.95 | 4.95 | 58.80 | 20.48 | 0.01 | 8.55 | 3.47 | 0.05 | 0.19 | 0.19 | 2.38 | 0.48 | 0.24 | 0.24 |
| In625 | 1.00 | 0.00 | 61.90 | 21.50 | 0.00 | 9.00 | 3.65 | 0.05 | 0.20 | 0.20 | 2.50 | 0.50 | 0.25 | 0.25 |

Below is a summary of the printing parameters of the layers in each sample.

**
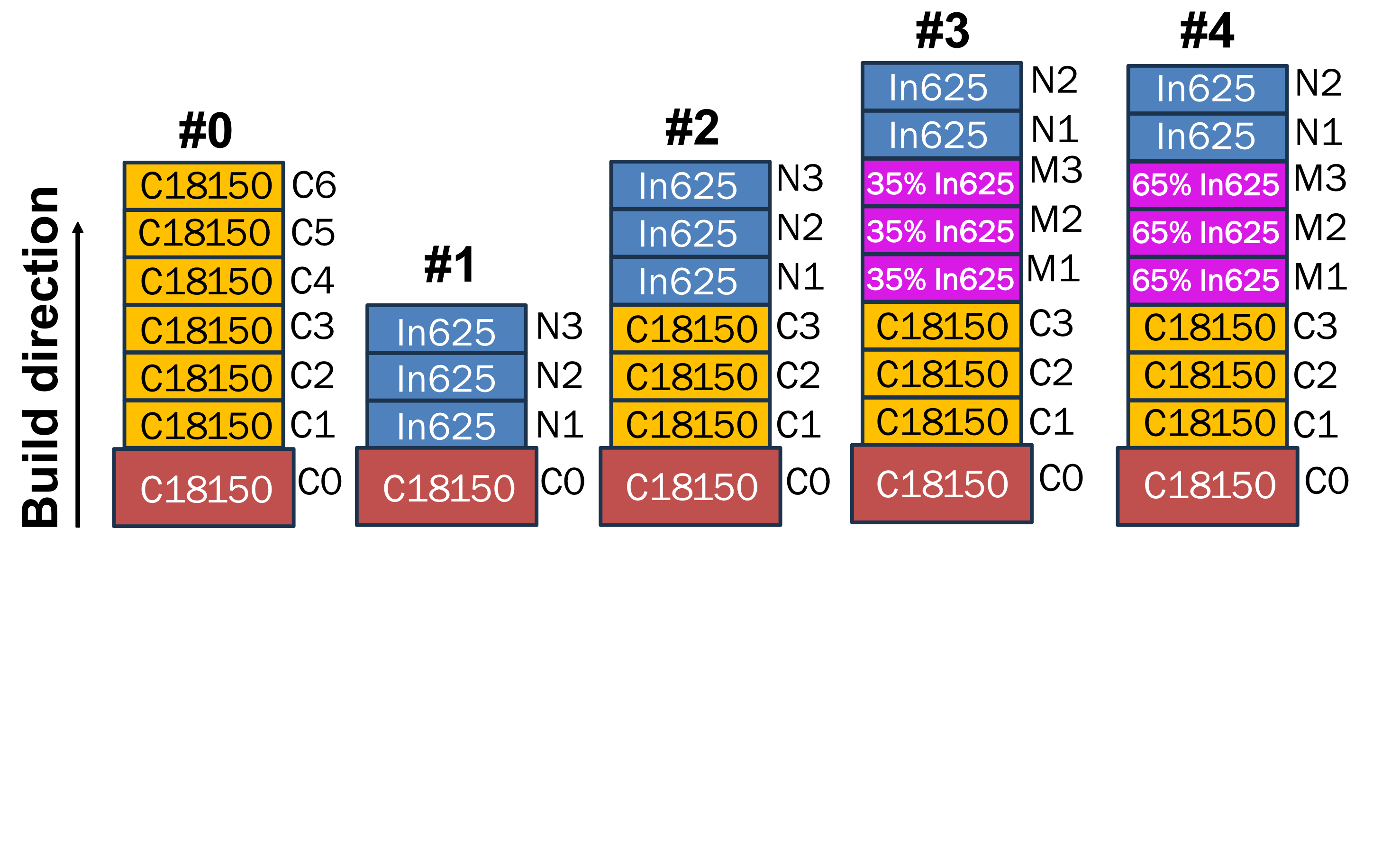
**

|  | Material | Travel speed (mm/s) | C18150 feed speed (m/min) | IN625 feed speed (m/min) | Current (A) |
| --- | --- | --- | --- | --- | --- |
| #0-C1 | C15180 | 4 | 2.49 | 0 | 250 |
| #0-C2 | C15180 | 4.5 | 2.49 | 0 | 250 |
| #0-C3~C6 | C15180 | 5 | 2.49 | 0 | 250 |
| #1-N1 | IN625 | 4 | 0 | 2.62 | 250 |
| #1-N2 | IN625 | 4.5 | 0 | 2.62 | 235 |
| #1-N3 | IN625 | 5 | 0 | 2.62 | 200 |
| #2-C1 | C15180 | 4 | 2.49 | 0 | 250 |
| #2-C2 | C15180 | 4.5 | 2.49 | 0 | 250 |
| #2-C3 | C15180 | 5 | 2.49 | 0 | 250 |
| #2-N1 | IN625 | 5 | 0 | 2.62 | 250 |
| #2-N2 | IN625 | 5 | 0 | 2.62 | 235 |
| #2-N3 | IN625 | 5 | 0 | 2.62 | 200 |
| #3-C1 | C15180 | 4 | 2.49 | 0 | 250 |
| #3-C2 | C15180 | 4.5 | 2.49 | 0 | 250 |
| #3-C3 | C15180 | 5 | 2.49 | 0 | 250 |
| #3-M1 | 35%IN625 | 5 | 1.62 | 0.92 | 250 |
| #3-M2 | 35%IN625 | 5 | 1.62 | 0.92 | 235 |
| #3-M3 | 35%IN625 | 5 | 1.62 | 0.92 | 215 |
| #3-N1 | IN625 | 5 | 0 | 2.62 | 215 |
| #3-N2 | IN625 | 5 | 0 | 2.62 | 215 |
| #4-C1 | C15180 | 4 | 2.49 | 0 | 250 |
| #4-C2 | C15180 | 4.5 | 2.49 | 0 | 250 |
| #4-C3 | C15180 | 5 | 2.49 | 0 | 250 |
| #4-M1 | 65%IN625 | 5 | 0.87 | 1.70 | 250 |
| #4-M2 | 65%IN625 | 5 | 0.87 | 1.70 | 235 |
| #4-M3 | 65%IN625 | 5 | 0.87 | 1.70 | 215 |
| #4-N1 | IN625 | 5 | 0 | 2.62 | 200 |
| #4-N2 | IN625 | 5 | 0 | 2.62 | 200 |

The following figure shows the layer stacking, EDS line scan, and BSE microstructure of sample #0~#4.


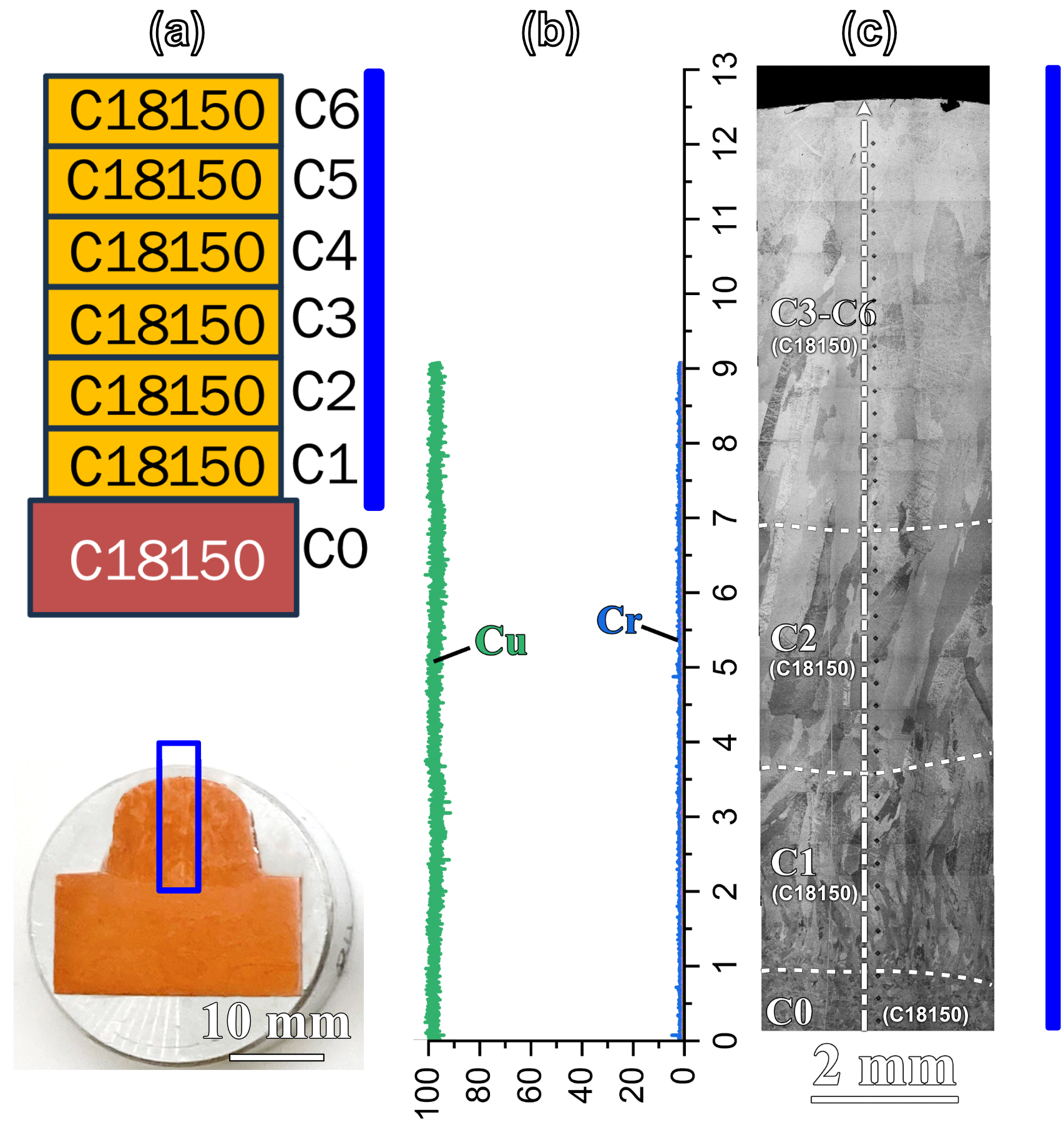


***Figure S2***. (a) Layer stacking, (b) EDS line scan, and (c) BSE microstructure of sample #0. The Blue marked area is observed by EDS and BSE. There are no cracks or phase separation found in the sample.


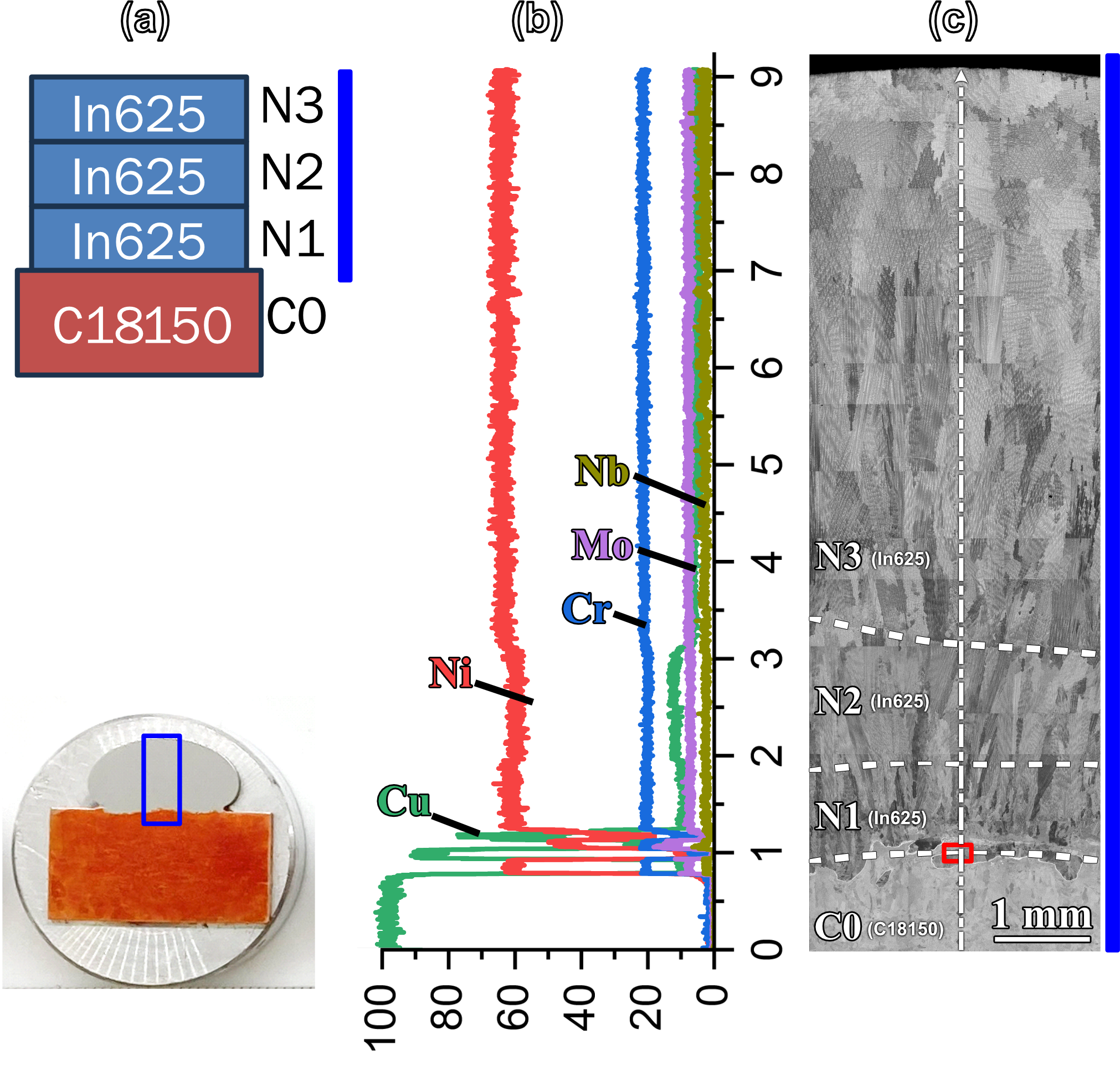


***Figure S3***. (a) Layer stacking, (b) EDS line scan, and (c) BSE microstructure of sample #1. The Blue marked area is observed by EDS and BSE. The red rectangle in (c) corresponds to the crack position in Figures 4 (a-d).


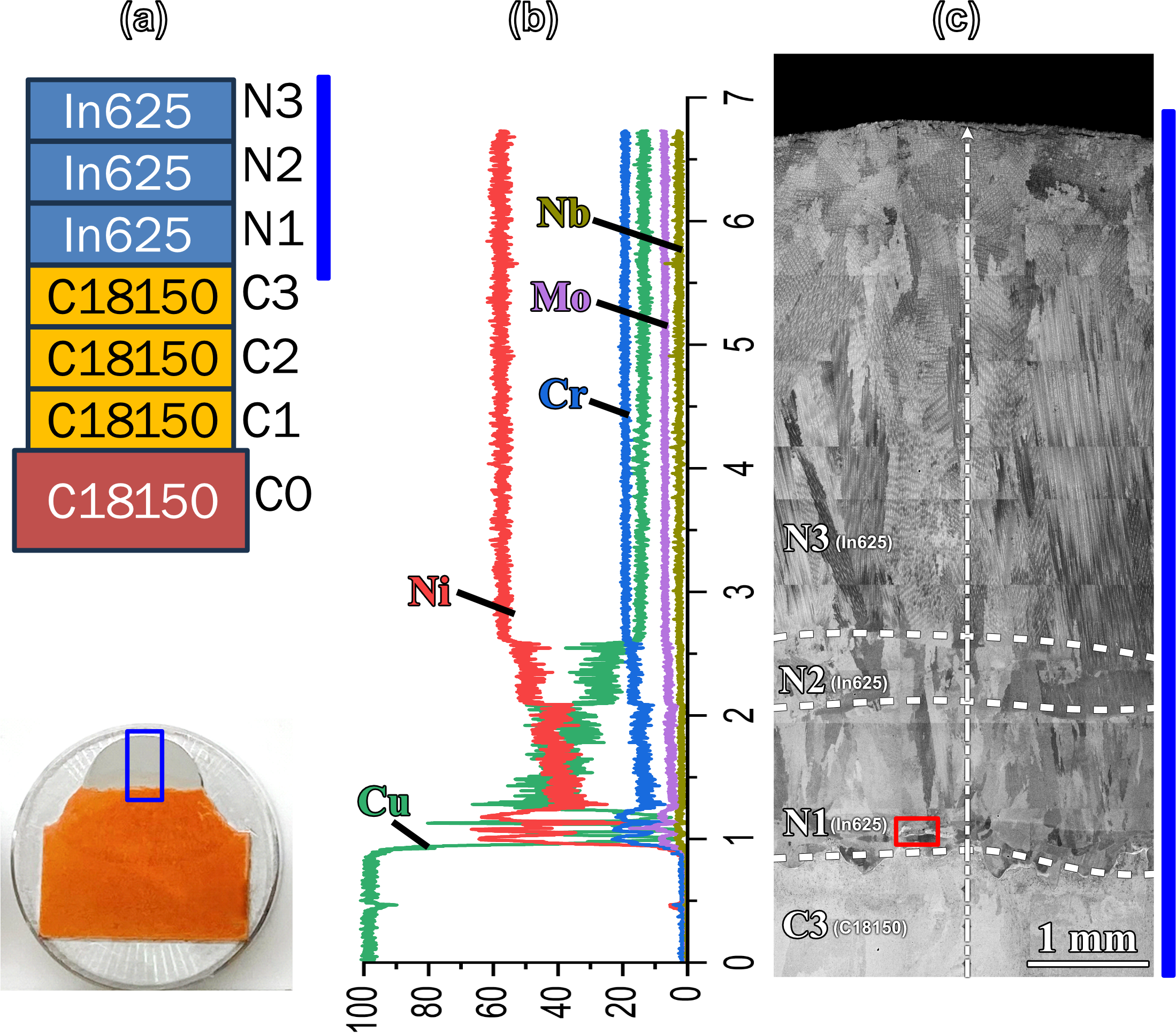


***Figure S4***. (a) Layer stacking, (b) EDS line scan, and (c) BSE microstructure of sample #2. The Blue marked area is observed by EDS and BSE. The red rectangle in (c) corresponds to the crack position in Figures 4 (e-h).


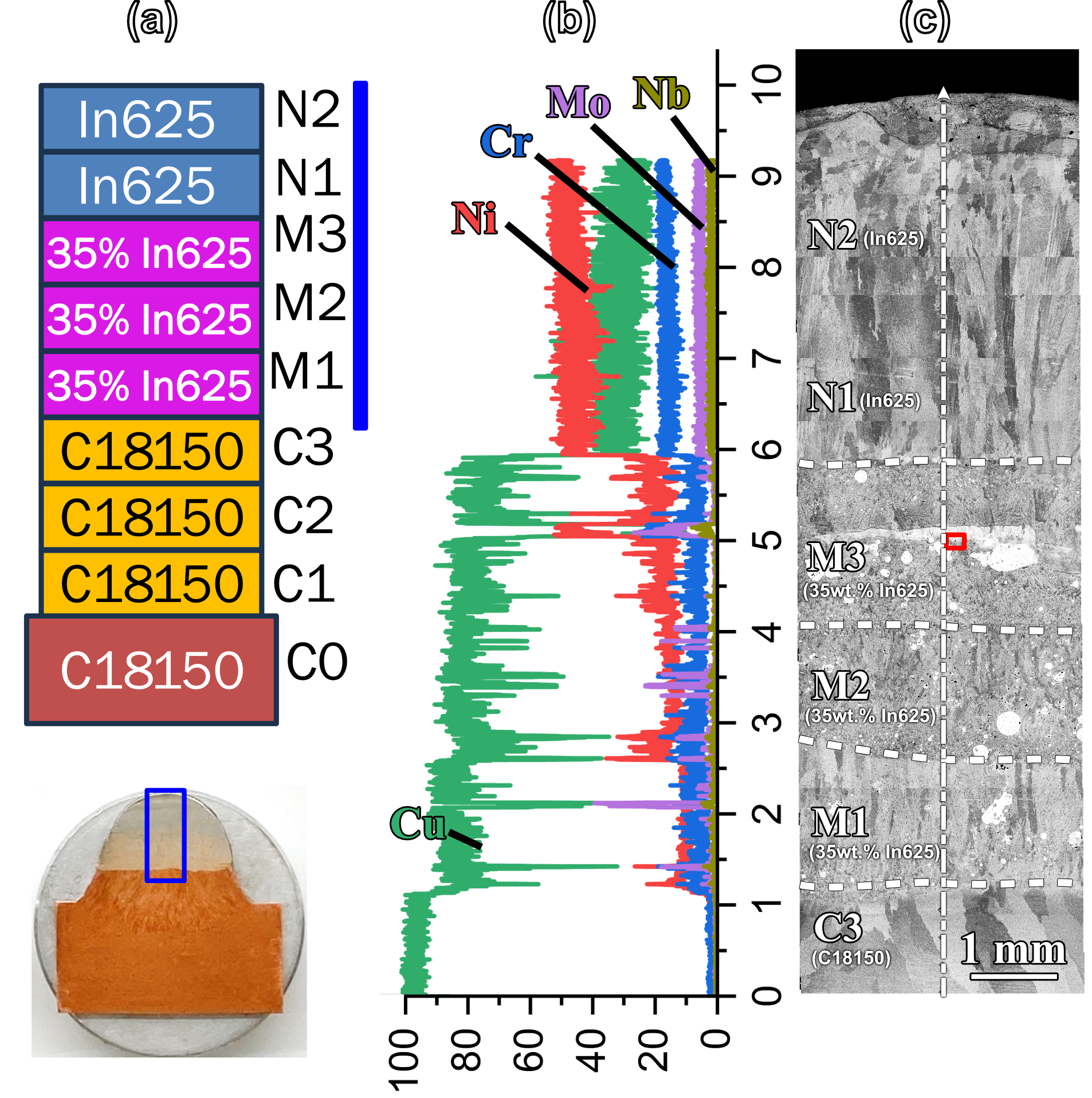


***Figure S5***. (a) Layer stacking, (b) EDS line scan, and (c) BSE microstructure of sample #3. The Blue marked area is observed by EDS and BSE. The red rectangle in (c) corresponds to the crack position in Figures 4 (i-l).


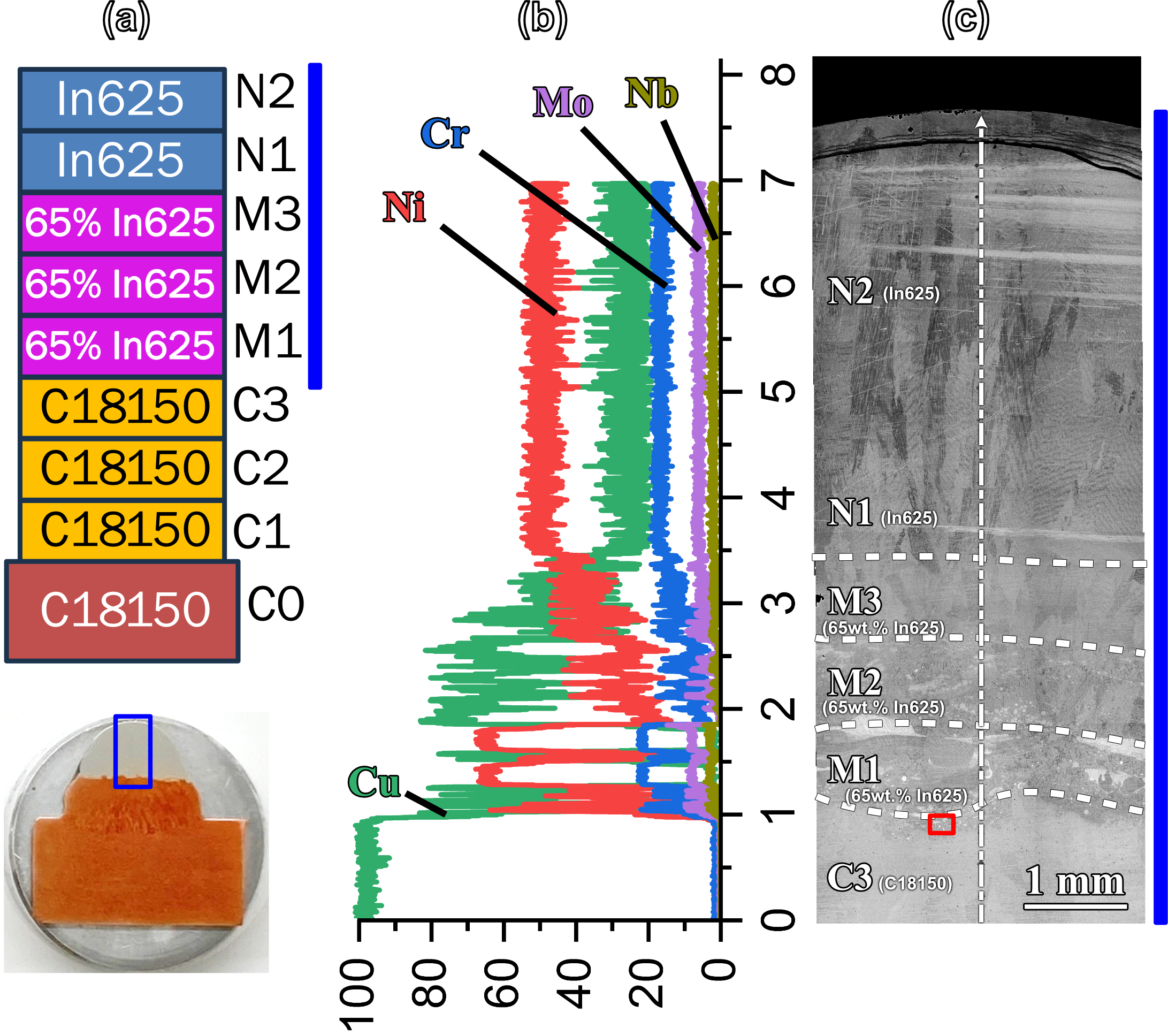


***Figure S6***. (a) Layer stacking, (b) EDS line scan, and (c) BSE microstructure of sample #4. The Blue marked area is observed by EDS and BSE. The red rectangle in (c) corresponds to the crack position in manuscript Figures 4 (m-p).
